# Renal Involvement in Granulomatosis With Polyangiitis Increases Economic Health Care Burden: Insights From the National Inpatient Sample Database

**DOI:** 10.7759/cureus.12515

**Published:** 2021-01-05

**Authors:** Osahon N Idolor, Armaan Guraya, Chukwudi C Muojieje, Sandhya Shri Kannayiram, Karun M Nair, Jesse Odion, Eseosa Sanwo, Osaigbokan P Aihie

**Affiliations:** 1 Internal Medicine, College of Medicine, University of Benin, Benin, NGA; 2 College of Osteopathic Medicine, Midwestern University Chicago, Chicago, USA; 3 Internal Medicine, Mountain View Regional Medical Center, Las Cruces, USA; 4 Internal Medicine, John H. Stroger, Jr. Hospital of Cook County, Chicago, USA; 5 Internal Medicine, University of Benin Teaching Hospital, Benin, NGA; 6 College of Medicine, University of Benin, Benin, NGA; 7 School of Medicine, University of Missouri, Columbia, USA

**Keywords:** granulomatosis with polyangiitis, renal, chronic kidney disease, vasculitis, mortality, national inpatient sample

## Abstract

Background

This study aims to compare outcomes of hospitalizations of granulomatosis with polyangiitis (GPA) with and without renal involvement. The primary outcome was inpatient mortality, whereas secondary outcomes were hospital length of stay (LOS) and total hospital charge.

Methods

Data were abstracted from the National Inpatient Sample (NIS) 2016 and 2017 databases. The NIS was searched for GPA hospitalizations with and without renal involvement as the principal or secondary diagnosis using International Classification of Diseases, Tenth Revision, Clinical Modification (ICD-10) codes. GPA hospitalizations for adult patients from the above groups were identified. Multivariate logistic and linear regression analyses were used to adjust for possible confounders for the primary and secondary outcomes, respectively.

Results

There were more than 71 million discharges included in the combined 2016 and 2017 NIS database, of which 23,670 were for adult patients who had either a principal or secondary ICD-10 code for GPA, and 8,265 (34.92%) of these GPA hospitalizations had renal involvement.

Hospitalizations for GPA with renal involvement had similar inpatient mortality (3.8% vs. 3.7%; adjusted OR: 1.14; 95% CI: 0.84-1.56; p=0.406) compared to those without renal involvement. GPA with renal involvement hospitalizations had an increase in adjusted mean LOS of 1.36 days (95% CI: 0.82-1.91; p=0.0001) compared to those without renal involvement. GPA with renal involvement hospitalizations had an increase in adjusted total hospital charges of $18,723 (95% CI: 9,595-27,852; p=0.0001) compared to those without renal involvement.

Conclusions

GPA with renal involvement hospitalizations had similar inpatient mortality compared to those without renal involvement. However, LOS and total hospital charges were greater in those with renal involvement.

## Introduction

Granulomatosis with polyangiitis (GPA), formerly known as Wegener's granulomatosis, is a rare vasculitis affecting the small- and medium-sized vessels. It is a type of antineutrophil cytoplasmic antibody (ANCA) associated vasculitis, most commonly affecting the upper and lower respiratory tract, the kidneys, and the eyes [1]. The incidence in the United States is three per one million population, with peak incidences at 64 to 75 years of age, and recent studies have shown no sex predilection [2]. Renal involvement is very common and, together with pulmonary involvement, represents the most severe complications of the disease [3-5].

The most common renal manifestation of GPA is rapidly progressive glomerulonephritis (RPGN), leading to chronic kidney disease (CKD) or end-stage renal disease (ESRD) [4-6]. Renal involvement is noted in only 10-20% at presentation, but 80% of patients eventually develop renal involvement within two years of disease onset [2,4]. RPGN is characterized clinically by a rapid decrease in the glomerular filtration rate (GFR) of at least 50% over a short period - from a few days to three months - and histologically pauci-immune necrotizing and extensive glomerular crescent formation. Other features include microscopic hematuria, often with erythrocyte casts, and usually non-nephrotic proteinuria (less than 3 g) [1,4,7]. Renal involvement is of particular importance because of its impact on prognosis [2,4].

Most prior studies have focused on the severity and impact of renal disease in GPA in terms of long-term prognosis as well as the clinical and laboratory associations of renal involvement in GPA. Several investigators have analyzed the impact of vasculitis and demographic features at the time of diagnosis on mortality among patients with GPA. However, there are few studies elaborating on the impact of renal involvement on the overall survival in patients with GPA [8].To bridge the gap in knowledge, this study aims to compare outcomes of hospitalizations of GPA with and without renal involvement using data abstracted from the National Inpatient Sample (NIS) database.

## Materials and methods

Data source

The NIS was searched for GPA hospitalizations with and without renal involvement using International Classification of Diseases, Tenth Revision, Clinical Modification (ICD-10) codes "M31.31" and "M31.30", respectively, as the principal or secondary diagnosis. NIS is a property of the Agency for Healthcare Research and Quality. It is the largest public inpatient database in the United States of America [9-13]. NIS is a 20% probability sampling across different strata, which is designed to be representative of all acute care hospitalizations in the US [14]. NIS maintains national representation by containing weighted discharges [15]. Each hospitalization in NIS 2016 can contain up to 30 ICD-10 diagnosis codes and 40 codes for NIS 2017. Diagnosis is either the principal diagnosis or secondary diagnosis. A principal diagnosis is the major ICD-10 code for admission. Any other diagnoses other than the principal diagnosis are secondary diagnoses [16]. This study was exempted from Institutional Review Board approval, as NIS contains de-personalized, publicly available patient data.

Inclusion criteria

We included all hospitalizations for adult patients ≥ 18 years of age. We used ICD-10 codes to identify principal/secondary diagnoses. See the Appendix for a complete list of ICD-10 codes used.

Outcomes

The primary outcome was inpatient mortality. Hospital length of stay (LOS) and total hospital charges were secondary outcomes of interest.

Statistical analysis

Analyses were performed using STATA Version 16 (StataCorp, College Station, TX, USA). A univariate logistic regression analysis using all variables and co-morbidities (Table 1) was used to calculate unadjusted odds ratios (ORs) for the primary outcome. All variables with p<0.1 were included in a multivariate logistic regression model. P-values < 0.05 were considered significant in the multivariate analysis. Literature review was used to select confounders. Charleston index was used to control for comorbidity complexity. Multivariate logistic and linear regression model with all variables and co-morbidities shown in Table 1 were used accordingly to adjust for confounders for the secondary outcomes.

## Results

There were more than 71 million discharges included in the combined 2016 and 2017 NIS database, of which 23,670 hospitalizations were for adult patients who had either a principal or secondary ICD 10 code for GPA. Of these hospitalizations, 8,265 (34.92%) and 15,405 (65.08%) were for GPA with renal and without renal involvement, respectively. GPA with renal hospitalizations had less females, more secondary diagnosis of congestive heart failure (CHF), CKD, maintenance hemodialysis, anemia, first to second quartile for expected income for zip code, Medicare insured, and Charleston comorbidity score ≥ 3 compared to GPA with renal involvement (Table 1).

**Table 1:** Baseline characteristics of granulomatosis with polyangiitis with and without renal involvement hospitalizations *Co-morbidities or secondary diagnoses GPA, granulomatosis with polyangiitis; MI, myocardial infarction; COPD, chronic obstructive pulmonary disease; DM, diabetes mellitus; CHF, chronic congestive heart failure; CKD, chronic kidney disease; O2, oxygen; median household income, median household income for patient’s Zip code

Variables	GPA (n=23,670)		
	Without Renal Involvement (n=15,405)	With Renal Involvement (n=8,265)	p-Value
Mean age (years)	62.23	61.82	0.461
Female	56.48%	47.01%	<0.0001
Race			
White	78.19%	74.31%	0.0975
Black	6.67%	7.77%	
Hispanic	10.38%	11.99%	
Asian	1.48%	1.77%	
Native Americans	1.15%	1.77%	
Others	2.12%	2.40%	
Charleston comorbidity index			
0	18.31%	7.32%	<0.0001
1	19.25%	5.38%	
2	18.47%	24.5%	
≥3	43.98%	62.79%	
Hospital bed size			
Small	16.52%	16.64%	0.8511
Medium	27.8%	26.98%	
Large	55.66%	56.38%	
Hospital teaching status			
Nonteaching	28.95%	28.68%	0.8507
Teaching	71.05%	71.32%	
Hospital location			
Rural	7.04%	6.84%	0.7929
Urban	92.96%	93.16%	
Expected primary payer			
Medicare	62.37%	66.67%	0.0403
Medicaid	9.18%	8.75%	
Private	26.47%	22.92%	
Self-pay	1.99%	1.66%	
Median household income (quartile)			
1st (0-25th)	23.87%	26.24%	0.0085
2nd (26th-50th)	25.75%	28.46%	
3rd (51st-75th)	25.95%	24.95%	
4th (76th-100th)	24.43%	20.34%	
Hospital region			
Northeast	20.16%	18.75%	0.2247
Midwest	25.22%	25.11%	
South	35.74%	34.66%	
West	18.89%	21.48%	
Co-morbidities*			
Dyslipidemia	35.02%	33.45%	0.2884
Old MI	5.71%	5.69%	0.9728
Atrial fibrillation/flutter	15.35%	16.45%	0.3274
COPD	22.85%	18.33%	0.0004
Carotid artery disease	0.75%	0.60%	0.5778
Old stroke	6.56%	6.35%	0.7961
Hypertension	29.99%	13.13%	<0.0001
Peripheral vessel disease	97.18%	2.66%	0.7493
Hypothyroidism	17.27%	16.52%	0.5197
DM type 1 and 2	25.90%	23.17%	0.0404
Obesity	15.64%	13.73%	0.0890
CHF	19.15%	25.29%	<0.0001
CKD	40.64%	79.79%	<0.0001
Liver disease	4.35%	3.09%	0.0402
Maintenance hemodialysis	12.33%	33.58%	<0.0001
O_2_ dependence	7.59%	5.02%	0.0009
Smoking	28.59%	28.61%	0.9889
Anemia	44.43%	71.51%	<0.0001

Inpatient mortality occurred in 1,010 (5.14%) GPA hospitalizations, of which 425 (3.80%) of the deaths occurred in GPA with renal involvement vs. 585 (3.70%) without renal involvement. The adjusted odds ratio of inpatient mortality for GPA with renal compared to without renal involvement was 1.14 (95% CI: 0.84-1.56; p=0.406). Mean LOS of hospitalization for GPA with renal involvement was 8.14 vs. 6.59 days for GPA without renal involvement. GPA with renal involvement hospitalizations had a mean increase in adjusted mean LOS of 1.36 days (95% CI: 0.82-1.91; p<0.0001) compared to GPA without renal involvement. Total hospital charge for GPA with renal involvement was $102,007 vs. $76,439 for GPA without renal involvement. GPA with renal involvement hospitalizations had an increase in adjusted total hospital charges by $18,723 compared to GPA without renal involvement (95% CI: 9,595-27,852; p<0.0001). See Table 2 for details.

**Table 2:** Clinical outcomes of granulomatosis with polyangiitis hospitalizations with and without renal involvement *Statistically significant. GPA, granulomatosis with polyangiitis; LOS, hospital length of stay; USD, United States Dollars

Primary Outcome	GPA with Renal Involvement	GPA without Renal Involvement	Adjusted Odds Ratio (95% CI)	p-Value
In-hospital mortality, %	3.80	3.70	1.14 (0.84-1.56)	0.406
Secondary Outcomes	GPA with Renal Involvement	GPA without Renal Involvement	Adjusted Mean Difference (95% CI)	p-Value
LOS, mean, days	8.14	6.59	1.36 (0.82-1.91)	<0.0001*
Total charge, mean, USD	102,007	76,439	18,723 (9,595-27,852)	<0.0001*

## Discussion

This study compared the outcomes of hospitalizations of GPA with and without renal involvement; 35% of hospitalized patients with GPA had renal involvement, whereas 65% had no renal involvement. According to Sinico et al., renal involvement in GPA occurs only in 10-20% of cases at presentation, but 80% of patients eventually develop renal vasculitis within two years of disease onset. However, the NIS database report does not indicate when renal involvement occurred. Patients with renal involvement in this study had more baseline comorbidities (CKD, CHF, anemia, and maintenance hemodialysis). Multiple studies have shown that the principal negative predictors for GPA patient survival are age >50 years, dialysis dependence at presentation, high creatinine in the first month, kidney involvement (with impaired renal function), and pulmonary manifestations at diagnosis [4,7,8,17,18]. The identification of prognostic factors is a crucial element for the clinician in balancing the risks against the treatment benefits [7]. Therapy is associated with severe and potentially lethal adverse effects for many patients; nearly 90% of patients experience persistent morbidity despite adequate treatment [19]. Therefore, there is a need for prognostic markers of renal outcome to help to modify therapy for patients who have ANCA-associated vasculitis such as GPA [19].

RPGN, the most common renal involvement in GPA, can lead to CKD or ESRD. The classical presentation is characterized by an RPGN, macroscopic or microscopic hematuria, proteinuria, edema, decreased urine output, and rapid progressive deterioration of renal function; even with appropriate therapy, the disease may lead to chronic renal failure [4,20,21]. Hence, up to 30% of patients with moderate-to-severe renal disease at the time of diagnosis will require renal replacement therapy such as hemodialysis. Between 40% and 70% of patients may recover renal function following induction therapy [21]. Factors associated with return to normal renal function are unclear. However, it depends on early therapy and high doses of immunomodulators [17,20,21]. Our study showed that a significant number of patients with renal involvement had CKD (79.79%) and that 34% of GPA patients with renal involvement required maintenance dialysis compared to 12% in those without renal involvement.

The fact that renal involvement is a determinant of poor long-term prognosis was demonstrated for the first time by Carrington and Liebow [17,22]. This finding has been supported by many other studies [4,5,17,22]. These studies demonstrated that only those patients whose renal involvement was associated with functional impairment were at a greater risk of death. These findings suggest that the prognosis might not depend on glomerulonephritis itself but on renal function deterioration [22]. However, our study did not show a significant increase in inpatient mortality in patients with renal disease. Nationwide trends in hospitalizations and in-hospital mortality of GPA over the past two decades are mostly unknown [5,22].

Early and late-onset comorbidities significantly impact the quality of life, morbidity, and mortality rates. The latter is still unsatisfactory, and cardiovascular complications are among the principal complications leading to death [5]. The patients with renal involvement in our study had a higher percentage of congestive heart failure (25.29%) compared to those without renal involvement (19.15%). GPA has been suggested to be a strong independent risk factor for heart failure, although scarce data exist regarding the prevalence of cardiovascular risk factors and outcomes, including heart failure. Cardiac involvement was first described by Wegner in 1936, and since then it has been reported in 6-44% of patients diagnosed with GPA, with the latter attributed to those with greater disease severity [19,23]. Alongside coronary arteritis, pericarditis is the most common cardiac manifestation (accounting for around 50% of all cardiac cases). Other cardiac presentations include ischemia, heart failure, valvular disorders, conduction abnormalities, and myocarditis [19,23].

We saw a significant increase in the mean length of hospital stay of 8.14 days for GPA with renal involvement compared to 6.59 days for GPA without renal involvement. GPA with renal involvement hospitalizations had an increase in adjusted mean LOS of 1.36 days (95% CI: 0.82-1.91; p<0.0001) compared to GPA without renal involvement. This is an interesting finding. There are no studies known to the authors to compare or contrast this finding. However, the study conducted by Luo et al., which investigated the 30-day all-cause hospital readmissions in patients with GPA using data from the 2014 National Readmission Database (NRD), a U.S.-based nationwide all-payer hospital inpatient database, is worthy of note. GPA readmissions were associated with higher LOS (8.0 vs. 7.2 days; p=0.019) and less discharge home (50% vs 63%, p<0.001) [24]. Hence GPA admissions with renal involvement and GPA readmissions prolong hospital LOS compare to GPA admissions without renal involvement and index GPA hospitalizations. The prolonged LOS in GPA with renal involvement admissions may indicate a higher level of complexity in these patients, which causes increased health care expenditure.

GPA with renal involvement hospitalizations had an increase in adjusted total hospital charges of $18,723 compared to GPA without renal involvement (95% CI: 9,595-27,852; p<0.0001). Studies available only compared hospitalization cost and charges of GPA hospitalizations vs. non-GPA hospitalizations. GPA hospitalizations were associated with higher healthcare expenditure as demonstrated by increased adjusted mean total hospital cost of $5,125 (95% CI: 4,719-5,531) and adjusted mean total hospital charges of $16,841 (95% CI: 15,280-18,403) compared to hospitalizations without GPA [25].

The large sample size that increases the study power is the major strength of our study. However, our study has some limitations. NIS uses claims data based on ICD-10 codes, which were created for billing purposes [26]. ICD-10 codes do not grade severity [27]; therefore, we cannot discern if GPA disease severity may have had affected outcomes. NIS database contains reports on hospitalizations rather than individual patients [28]. Data on medication compliance is not available in the NIS [29,30]. NIS does not contain information on the time of diagnosis and duration of renal involvement in GPA patients.

## Conclusions

There is no statistically significant difference in inpatient mortality for hospitalizations of GPA with and without renal involvement. However, LOS and total hospital charge in GPA with renal involvement were greater than those without renal involvement. Hence, GPA with renal involvement has a greater burden to the healthcare system compared to without renal involvement.

## Appendices

**Table 3:** Supplementary table of used ICD-10 codes ICD-10, International Classification of Diseases, Tenth Revision; GPA: granulomatosis with polyangiitis, MI: myocardial infarction, COPD, chronic obstructive pulmonary disease, DM: diabetes mellitus, CKD, chronic kidney disease, O_2_, oxygen

	ICD-10 codes
Diagnosis codes	
GPA with renal involvement	M31.31
GPA without renal involvement	M31.30
Comorbidities	
Dyslipidemia	E78
Old MI	I252
Atrial fibrillation/flutter	I48
COPD	J41, J42, J43, J44
Carotid artery disease	I652
Old stroke	I63
Hypertension	I10
Peripheral vascular disease	I739
Hypothyroidism	E03
DM type 1 and 2	E10, E11
Obesity	E660, E6601, E6609, E661, E662, E668, E669
Congestive heart failure	I50
CKD	N18
Liver disease	K70, K71, K72, K73, K74, K75, K76, K77
Maintenance dialysis	Z992
O_2_ dependence	Z9981
Smoking	Z87891, F17200
Anemia	D50, D51, D52, D53, D55, D56, D57, D58, D59, D60, D61, D62, D63, D64
